# Atypical Asymmetric Presentation of Severe Graves’ Orbitopathy

**DOI:** 10.7759/cureus.45907

**Published:** 2023-09-25

**Authors:** Priyanka Gupta, Navdeep Kaur, Aman Goyal, Anupriya Aggarwal

**Affiliations:** 1 Department of Ophthalmology, Adesh Institute of Medical Sciences and Research, Bathinda, IND

**Keywords:** graves' disease, graves' orbitopathy, extra-ocular muscle hypertrophy, orbital apex syndrome, thyroid-associated orbitopathy

## Abstract

Graves' disease is a self-limiting autoimmune thyroid disorder caused by stimulating antibodies to the thyroid-stimulating hormone receptor. It usually affects middle-aged females in the fourth to sixth decade of life. It is distinguished by keratopathy, chemosis, proptosis, and eyelid swelling, in addition to ocular discomfort. A total of 3-5% of cases present with a severe form of Graves' orbitopathy, which manifests with diminution of vision, optic nerve compression, optic neuropathy, and exposure keratopathy. We describe a case of a 34-year-old female patient who presented with the chief complaint of rapid deterioration of vision over a period of three months in the right eye. Ocular examination revealed proptosis, widened palpebral aperture, elevation of intra-ocular pressure (IOP) in the upgaze, restricted eye movements, and signs of optic nerve compression. Findings were confirmed on a CT scan of the orbit. The unusual presentation in this case was that she had rapid, significant deterioration of vision in the right eye, with a progression of proptosis more marked in the contralateral eye. This underlies the importance of thoroughly examining for any possible orbital apex syndrome in both eyes, not just the eye with marked proptosis. The patient, being reluctant for orbital decompression, was prescribed IV methylprednisolone 1 g for three consecutive days, which reduced her proptosis and improved her vision. This acted as a temporary measure to increase the duration of the surgical window until the time the patient undergoes the surgery.

## Introduction

Graves’ disease is a self-limiting autoimmune thyroid disorder caused by stimulating antibodies to the thyroid-stimulating hormone receptor (TSH-R) on thyroid follicular cells, causing hyperthyroidism. It occurs in 20-30 cases per 100,000 population. It usually affects middle-aged females in the fourth to sixth decade of life with a history of smoking, life stressors, and a history of poorly controlled hypothyroidism following radioactive iodine treatment [1]. In addition, there is a family history of the same, which increases the risk of the development of a severe form of Graves’ disease [1]. It can present with various ocular manifestations like proptosis, exposure keratopathy, diminision of vision, and diplopia, which indicate a severe form of Graves’ orbitopathy. A sudden, recent increase in any of the above presentations indicates that the disease is in the active phase. A total of 3-5% of cases that present with a severe form of Graves' orbitopathy may manifest with diminision of vision, optic nerve compression, or corneal ulceration [2,3].

Compressive optic neuropathy (CON) is a rare vision-threatening condition caused by compression of the optic nerve at the orbital apex by hypertrophied muscles that reportedly occurs in 5-6% of patients with severe thyroid-associated ophthalmopathy [4]. Herein, we shall delve deeper into the clinical course of a patient with vision-compromising compressive optic neuropathy secondary to Graves’ disease.

## Case presentation

A 34-year-old female patient came to us with the chief complaint of rapid deterioration of vision over a period of three months in the right eye. Her best-corrected visual acuity (BCVA) was counting finger near face OD and 6/9 OS. She also complained of protrusion of both eyes, the left eye more than the right eye, for 11 years.

The patient was diagnosed as a case of primary hyperthyroidism at the age of 22 years and was prescribed carbimazole 20 mg twice a day. However, the patient was non-compliant with the medication. Three years down the lane, she noticed a swelling in her neck, which was accompanied by both eyes protrusion, the left eye more than the right. Both gradually increased in size over a period of 12 years. She was prescribed IV methylprednisolone thrice in the past for her increasing proptosis, although the patient didn’t adhere to the prescribed dosage and duration of the same.

When the patient presented to us, she reported a drop in her BCVA OD from 6/9 to counting finger near face (CFNF) over three months. Her detailed examination was done. In the right eye, color vision and contrast sensitivity could not be assessed given the poor vision, and in the left eye, color vision was normal and contrast sensitivity was 6%. IOP was measured using a Perkins applanation tonometer and was found to be 12 mm OD and 11 mm OS in straight gaze, which increased to 27 mm OD and 19 mm OS in up gaze.

**Figure 1:**
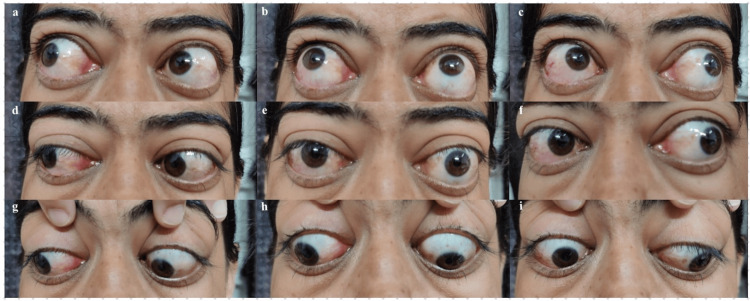
Ocular movement in all nine gazes showing varying degrees of restriction in each eye. The images show (a) -1 and -2 in OD and OS, (b) -2 in OU, (c) -3 and -1 in OD and OS, (d) -1 and -2 in OD and OS, (e) primary gaze, (f) -3 and -2 in OD and OS, (g) -1 and -2 in OD and OS, (h) -2 and 0 in OD and OS, (i) -3 and -1 in OD and OS.

**Figure 2:**
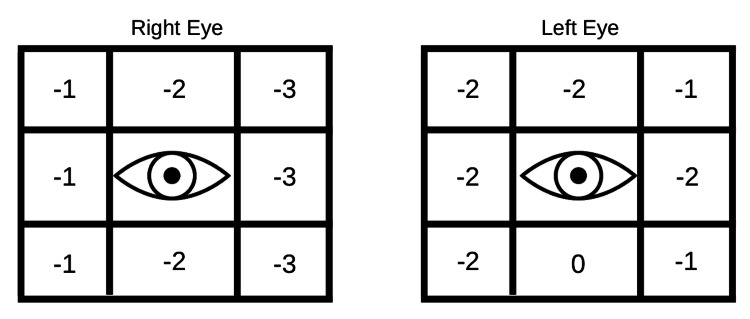
Extent of extra-ocular movement restriction.

Hertel’s exophthalmometer was used to measure her exophthalmos and was found to be 25 mm OD and 27 mm OS, which had increased considerably over the past three months from the previous values of 25 mm and 21 mm in OD and OS, respectively. So over the last three months, she has suffered an unusual and striking rapid deterioration of vision in the right eye, in contrast to rapid increase in proptosis in the left eye. The palpebral fissure height was 16 mm and 18 mm in OD and OS, respectively. Her extra-ocular movements were restricted in all the gazes, right eye more than left eye (Figures 1a-1i). The extent of extra-ocular movement restriction is demonstrated in Figure 2.

**Figure 3:**
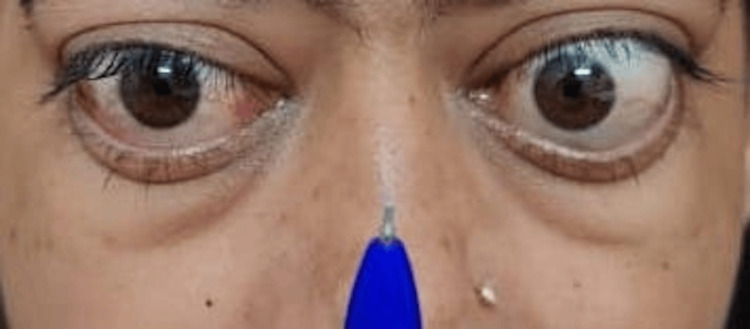
Conversion insufficiency - Mobius sign.

Her examination also yielded the presence of Mobius sign, Von Graefe’s sign (retarded descent of the upper lid on downgaze), Dalrymple’s sign (lid retraction in primary gaze), Enroth sign (edema of lower lid) (Figure 3). The right eye pupil had a sluggish and ill-sustained reaction to light, whereas the left eye pupil responded briskly to light. The rest of the anterior segment was unremarkable. Her clinical activity score (CAS) was found to be more than four. There was the presence of eyelid swelling, conjunctival redness, increase in proptosis of more than 2 mm, and decrease in visual acuity of more than 1 Snellen line.

**Figure 4:**
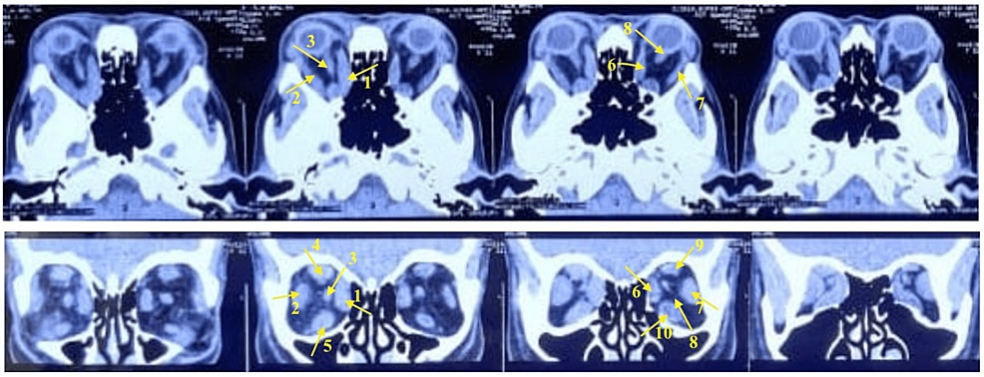
CT orbit showing both eye hypertrophy of extra-ocular muscles and retro-orbital fat stranding with orbital apex syndrome in the right eye. The images show (1 and 6) hypertrophied medial rectus (5.2 mm and 6.1 mm) in OD and OS, respectively; (2 and 7) hypertrophied lateral rectus (4.9 mm and 4 mm) in OD and OS, respectively; (4 and 9) hypertrophied superior rectus and LPS complex (11.8 mm and 8.6 mm) in OD and OS, respectively; (5 and 10) hypertrophied inferior rectus (9.22 mm and 11.2 mm) in OD and OS, respectively; and (3 and 8) optic nerve being compressed by the hypertrophied extra-ocular muscles in OD and OS, respectively.

Patient underwent Humphrey visual field analysis in the left eye, which showed constriction of peripheral visual fields. It was not performed in the right eye because of poor vision. The CT scan of the orbit revealed significant changes in muscle thickness of both eyes. The superior rectus muscle of the right eye exhibited a substantial 25% increase from the normal reference range, which was followed by growth in other muscles as follows: the inferior rectus by 19%, the lateral rectus by 15%, and the medial rectus by 11%. Similarly, the left eye displayed a significant 23% enlargement in the inferior rectus, leading to subsequent growth in other muscles as follows: the superior rectus by 18%, the medial rectus by 15%, and the lateral rectus by 12% (Table 1) [5]. Apart from this, both eyes showed hypertrophy of the levator palpebrae superioris and oblique muscles. All the muscles had thickening of the belly with sparing of their tendinous insertions. Also seen was retro-orbital fat stranding (Figure 4).

**Table 1:** CT orbit showing extra-ocular muscle progression with thickness of each rectus muscle with reference to normal range.

Extra-ocular muscle (normal thickness range)	Superior rectus (3.2-6.1 mm)	Inferior rectus (3.2-6.5 mm)	Lateral rectus (1.7-4.8 mm)	Medial rectus (3.3-5.0 mm)
Right eye	11.8 mm (25%)	9.22 mm (19%)	4.9 mm (15%)	5.2 mm (11%)
Left eye	8.6 mm (18.4%)	11.2 mm (23%)	04 mm (12%)	6.1 mm (15%)

Her free T3 levels ranged between 7.64 and 159 pmol/L, her free T4 levels varied from 1.69 to 53.94 pmol/L, and her TSH levels varied from 0.0091 to 67.99 µIU/mL on different occasions due to poor compliance (Table 2).

**Table 2:** Thyroid profile values of the patient.

Variables	Free T3 (3.1-6.8 pmol/L)	Free T4 (12-22 pmol/L)	TSH (0.22- 4.4 µIU/mL)
7 months prior	23.30	53.94	53.94
5 months prior	159	7.39	0.0091
1 month prior	58.98	1.69	67.99
At presentation	7.64	17.84	15.81

The patient was advised to undergo right orbital decompression in view of severe compressive optic neuropathy which was causing a rapid decrease in her vision. The patient however denied the same. She was then put on inj. intravenous methylprednisolone (IVMP) 1 g for three consecutive days. On subsequent follow-ups done at 15 days and one-month intervals, her visual acuity improved to 5/60 OD and 6/9 OS, respectively. Her proptosis was also reduced to 23 mm and 25 mm in OD and OS, respectively. However, the patient is yet to undergo orbital decompression.

## Discussion

Thyroid orbitopathy in Graves’ disease has an incidence of 16 cases per 100,000 in females and 2.9 per 100,000 in males [6]. The usual diagnosis of Graves’ disease is often established based on clinical features, elevated levels of thyroxine (T4) and triiodothyronine (T3), and undetectable levels of TSH. Additional testing may include measuring TSH-receptor antibodies (TRAb), radioactive iodine (RAI) uptake, or thyroid ultrasound with Doppler, each of which can confirm the diagnosis of Graves’ disease. Such patients may present with ocular manifestations like proptosis, sudden rapid vision loss, elevated palpebral fissure height (PFH), and elevated intra-ocular pressure in upgaze in both eyes.

**Figure 5:**
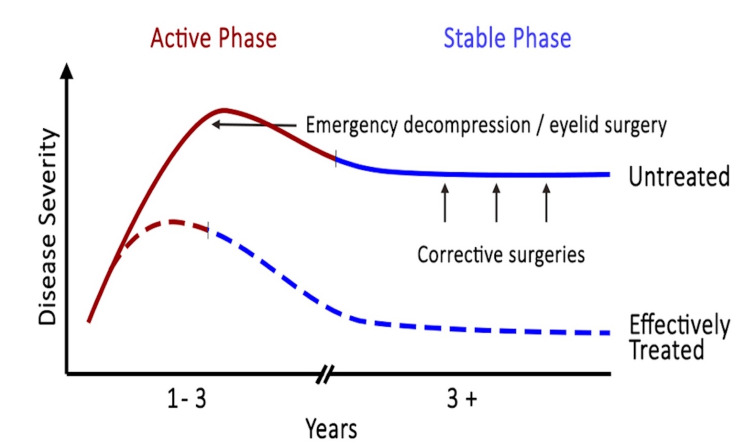
Rundle’s curve depicting active and stable phases of disease and the significance of early intervention in both stages of disease.

Rundle described the biphasic progression of thyroid-associated ophthalmopathy as having an active, dynamic phase with a mean duration lasting between 6 and 18 months and a dormant, static period [7]. Rundle’s curve is depicted in Figure 5.

The active disease occurs due to the activation of the immune process, which is represented by the pluripotential orbital fibrocytes found in orbital fat tissue and striated muscle. Thyrotropin-receptor antibodies (TRAB), also known as TSH-R, are secreted by an aberrant population of lymphocytes in Graves' disease, which is responsible for the manifestation of this disease. The circulating TRAB binds to CD40+ receptors expressed by the orbital fibrocytes and induces the upregulation of inflammatory cytokines such as IL-6, IL-8, and prostaglandin analog E2 (PGE2), which increases the accumulation of hyaluronic acid and glycosaminoglycan [1]. These get accumulated between muscle fibers, leading to muscle hypertrophy. Also seen in active thyroid-associated orbitopathy (TAO) is increased orbital fat volume. This is due to venous congestion from compression of the superior ophthalmic vein by the enlarged muscles and also due to intrinsic adipose inflammation. Both muscle hypertrophy and increased orbital fat volume contribute to proptosis in the active phase of TAO. The muscles also get infiltrated by inflammatory cells, such as lymphocytes, macrophages, plasma cells, and eosinophils. In long-standing cases, increased collagen deposition leads to muscle fibrosis.

The reference normal size range for the inferior rectus is 3.2-6.5 mm, medial rectus is 3.5-5.0 mm, superior rectus is 3.2-6.1 mm, and lateral rectus is 1.7-4.8 mm [5]. However, because of the mechanism stated above, the extra-ocular muscle size increases in TAO. The increase in the size of the muscle correlates with both the severity of the disease and the risk of optic nerve compression.

Another clinical feature associated with TAO in its severe form is compressive optic neuropathy. The hypertrophied muscles and increased orbital fat volume hinder axonal transport and signal transmission by squeezing the blood supply and inducing ischemia to the nerve or by directly inflicting a mass effect on the axons, thereby leading to CON. The sections of the nerve that travel through small bone structures, such as the orbital apex and optic canal, are those that are most prone to compression [8].

In our case, which was a severe variant of TAO, the patient had rapid progression of proptosis in the left eye and significant dimness of vision in the right eye over a three-month period. This was the active phase of the disease, wherein all the extra-ocular muscles underwent significant hypertrophy; the superior rectus and inferior rectus were involved more as compared to the medial and lateral rectus. The size of the muscles usually correlates with both the severity of the disease and the risk of optic nerve compression [9]. But this is contrary to our case, wherein both eyes of the patient had symmetrical enlargement of extraocular muscles, but proptosis was more marked in the left eye, and diminision of vision was more in the right eye. Therefore, we emphasize the role of increased orbital fat volume due to venous congestion and not just the muscle enlargement in the left eye, which had marked proptosis. The possible mechanism of diminution of vision being more pronounced in the right eye can be explained by higher localized orbital inflammation surrounding the optic nerve, leading to orbital apex syndrome. This is evident on the CT as retro-orbital fat stranding.

In similar case studies, thickening of extra-ocular muscles has been reported. A case study by Hirokawa et al. had hypertrophy of superior rectus and levator palpebrae superioris [4]. Another case reported by Yip et al. had hypertrophy of medial rectus muscles in both eyes and the superior and inferior rectus muscles in the left and right eye, respectively [2]. Verma et al. reported slight enlargement of the inferior, medial, and lateral recti muscles [10]. The case reported by El Othman et al. had edema in the right inferior and right lateral muscles, along with comparatively milder involvement of the left inferior orbital muscle [11]. However, in our case, all four recti and levator palpebrae superioris were hypertrophied, along with inflammation of the retro-orbital tissue.

The management of patients with thyroid-associated orbitopathy involves various approaches, depending on the severity of the condition. The treatment is preferably aimed at the early active inflammatory phase to lessen the burden of ocular disease as per the disease pattern depicted by Rundle's curve [7]. Antithyroid drugs, such as methimazole, carbimazole, and propylthiouracil, are used to normalize thyroid hormone levels and may lead to remission. Alternatively, radioactive iodine (RAI) therapy or surgical removal of the thyroid gland can be considered to destroy or remove the thyroid gland. Aggressive therapies are employed in severe forms, such as oral or intravenous glucocorticoids, rituximab, tocilizumab, and orbital radiotherapy.

However, if there are significant visual symptoms requiring immediate orbital decompression (such as compressive optic neuropathy, corneal decompensation, and extremely excruciating pain), surgical intervention is necessary in patients with active TED.

Steroids play a crucial role in controlling the inflammation associated with TAO. Compared to orally taken glucocorticoids, IVMP pulsed therapy is more successful and has fewer side effects as the first-line treatment for moderate-to-severe active Graves' orbitopathy. It might prevent irreversible eye damage. It functions by reducing inflammation, thereby reducing the edema of the orbit. However, IVMP can have negative side effects in some individuals, such as a hypercoagulable condition, which raises the risk of myocardial infarction and venous thromboembolism. As a result, it is recommended that individuals who are at a higher risk of developing venous thromboembolism undergo anticoagulation prophylaxis [12,13]. In our study, the patient presented with a sight-compromising condition and, despite counseling for orbital decompression, wanted to defer the surgery for some time. During this time, intravenous methylprednisolone helped to reduce the inflammation, thereby reducing proptosis and improving vision. In such cases, IV methylprednisolone can act as a savior to buy some time for patients reluctant to undergo surgery.

Teprotumumab, a novel antibody targeting the insulin-like growth factor-1 receptor (IGF-1R), has shown promise in the treatment of active Graves' ophthalmopathy. It plays an important role in both the acute and chronic phases of the condition and has demonstrated benefits in reducing proptosis, inflammation, diplopia, strabismus, and orbital soft tissue volume [1].

## Conclusions

Initiating therapy early is crucial to diminishing the overall severity of the disease. The absolute amount of proptosis is not predictive of visual outcome, as other factors like orbital apex compression are crucial in determining the final visual outcome. However, rapid progression in the proptosis or sudden deterioration of vision points to an active phase of disease, which needs to be managed aggressively.
